# Swipe, watch, learn? An analysis of TikTok as a source of patient education on spondylolisthesis

**DOI:** 10.1016/j.bas.2026.105960

**Published:** 2026-02-03

**Authors:** Celine Akta, Moses El Kayali, Lukas Schönnagel, Luis Bürck, Maximilian Muellner, Friederike Schömig, Matthias Pumberger, Tom Folkerts

**Affiliations:** Center for Musculoskeletal Surgery, Charité - Universitätsmedizin Berlin, Corporate Member of Freie Universität Berlin, Humboldt-Universität zu Berlin, and Berlin Institute of Health, Berlin, Germany

**Keywords:** Social media, TikTok, Spondylolisthesis, Spine surgery, Patient education, Digital health

## Abstract

**Introduction:**

Social media has become a major source of health information. TikTok, a rapidly expanding global platform that enables broad dissemination of medical content, yet the accuracy and reliability of such information remain uncertain. In this context, assessing the educational quality of videos on spondylolisthesis is of increasing clinical relevance.

**Research question:**

To evaluate the quality, reliability, and educational value of TikTok videos on spondylolisthesis and identify factors associated with higher-quality content.

**Material and methods:**

TikTok was searched in August 2025 using the keyword “spondylolisthesis.” Video metrics, uploader type and content category were recorded. Two orthopedic surgeons independently assessed reliability and quality using the DISCERN tool, Journal of the American Medical Association (JAMA) benchmarks, and Global Quality Score (GQS).

**Results:**

A total of 254 TikTok videos were screened, of which 82 met inclusion criteria, totaling 4.15 million views and 55,967 likes. Private users uploaded 46.3%, surgeons 28.0%, physiotherapists 23.2%, and researchers 2.4%. Overall quality was poor (DISCERN 34.1 ± 17.6; JAMA 1.8 ± 1.1; GQS 2.6 ± 1.1). Videos by surgeons and physiotherapists scored significantly higher (p < 0.001), and educational content outperformed patient experiences (p < 0.001). Longer videos correlated with higher quality scores, while engagement metrics were not predictive.

**Discussion and conclusion:**

Most TikTok videos on spondylolisthesis showed low quality and limited reliability. Educational content produced by healthcare professionals performed better, while popularity metrics were not indicative of quality. Spine specialists should recognize TikTok's growing role in patient education and contribute accurate, evidence-based content to improve information quality.

## Highlights

• TikTok videos on spondylolisthesis are largely low in quality and reliability.
• Videos created by surgeons and physiotherapists achieved higher quality ratings.
• Longer and educational videos showed higher overall quality and value.
• Engagement metrics (likes, views, shares) did not predict educational value.

## List of abbreviations

CI: Confidence Interval
CME: Continuing Medical Education
COPD: Chronic Obstructive Pulmonary Disease
CPD: Continuing Professional Development
GQS: Global Quality Score
JAMA: Journal of the American Medical Association
SD: Standard Deviation
VIF: Variance Inflation Factor

## 1 Introduction

Spondylolisthesis is a vertebral subluxation, characterized by the displacement of a vertebral body relative to the subjacent segment, most commonly in an anterior direction (Saremi et al., 2024; Rangwalla et al., 2024; Gallagher et al., 2020; Elmose et al., 2023). Degenerative lumbar spondylolisthesis, linked to progressive disc degeneration, and isthmic lumbar spondylolisthesis, resulting from a defect in the pars interarticularis, are the most prevalent types, whereas traumatic, dysplastic, and pathological variants occur less frequently (Saremi et al., 2024; Gallagher et al., 2020; Ver et al., 2019). Spondylolisthesis is a frequent cause of low back pain, radiculopathy, and neurogenic claudication (Elmose et al., 2023; Campbell et al., 2017; Martin et al., 2019). The presence of severe symptoms or progressive neurological deficits frequently warrants surgical treatment, rendering spondylolisthesis a major indication for lumbar spine surgery (Campbell et al., 2017; Martin et al., 2019; Fujimori et al., 2015; Spiker et al., 2019). Given the rising incidence of low back pain worldwide and increasing age of the population, the clinical importance of spondylolisthesis continues to expand (Spiker et al., 2019; Ravindra et al., 2018).

Over the past decade, social media has become a central medium for the dissemination of health-related information (Smailhodzic et al., 2016; Sylvia Chou et al., 2020; Rodrigues et al., 2024; Wharton et al., 2025). Platforms such as YouTube (Google LLC, Mountain View, Ca, USA), Instagram (Meta Platforms, Inc., Menlo Park, CA, USA), and Facebook (Meta Platforms, Inc., Menlo Park, CA, USA) allow rapid sharing of educational content to a global audience. While the accessibility of social media enables patients - particularly those with limited access to healthcare providers - to obtain medical guidance without direct clinical contact, it is accompanied by enduring concerns regarding the accuracy, quality, and reliability of the information disseminated (Sylvia Chou et al., 2020; O'Sullivan et al., 2022; Subramanian et al., 2023; Glover et al., 2025). Only a small proportion of health-related information on these platforms originates from qualified healthcare professionals, thereby circumventing traditional peer review (Subramanian et al., 2023; Samtani et al., 2023; Youssef et al., 2025). This issue is particularly relevant for complex spinal conditions such as spondylolisthesis, where precise knowledge of diagnosis, treatment options, and surgical indications is crucial for informed decision-making (Martin et al., 2019; Steiger et al., 2014).

While these challenges are shared across social media, TikTok (ByteDance Ltd., Beijing, China), a short-form video platform, has emerged as the most prominent example, experiencing exponential growth since its inception in 2016, now surpassing 1.6 billion active users and 5 billion downloads worldwide (Sylvia Chou et al., 2020; TikTok Revenue and Usage Statistics, 2025; Yaradılmış et al., 2020; D'Ambrosi et al., 2025). Driven by its highly engaging algorithmic feed, TikTok allows for the rapid and extensive dissemination of medical information (Sylvia Chou et al., 2020). Given TikTok's popularity and its increasing influence on patient perceptions, evaluating the quality of educational videos related to spondylolisthesis on this platform is of critical importance.

To date, however, no study has focused on evaluating TikTok as a source of patient education for spondylolisthesis. The present analysis addresses this gap by systematically assessing the quality, reliability, and educational value of TikTok videos related to spondylolisthesis. Furthermore, it aims to identify characteristics of higher-quality content, thereby informing strategies to improve future medical videos on TikTok and enabling patients to distinguish reliable, evidence-based content from misleading or low-value information.

## 2 Materials and methods

### 2.1 Identification and selection of videos

**Fig. 1:**
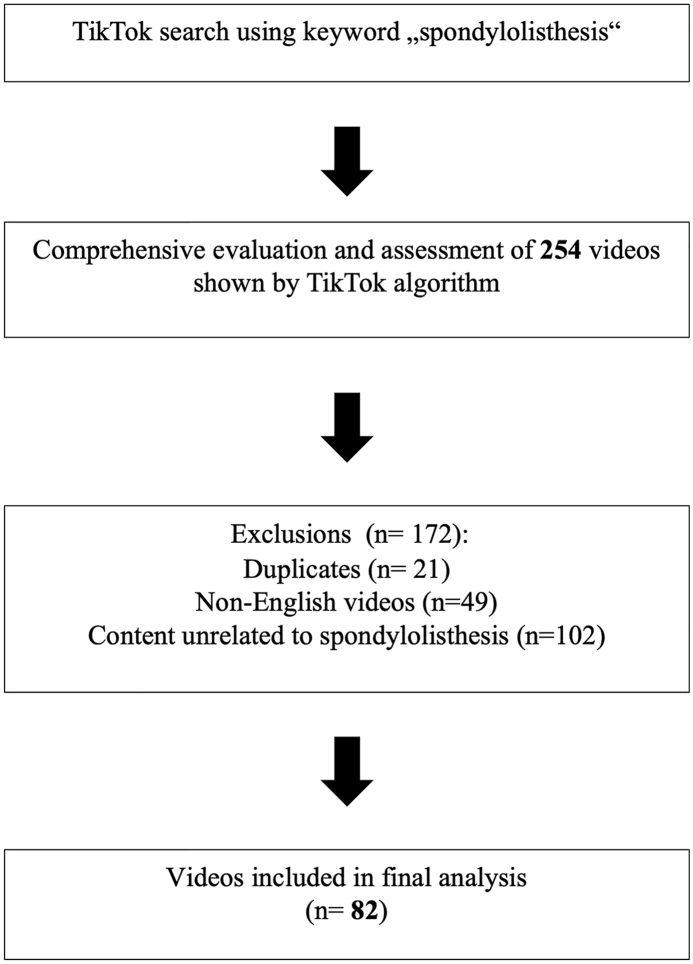
Flow chart of TikTok video selection process.

The social media platform TikTok (https://www.tiktok.com) was searched on August 11, 2025, to identify videos related to spondylolisthesis. TikTok videos were identified using the keyword “spondylolisthesis”. The search was conducted using a newly created account with no prior search history, thereby approximating the content most likely encountered by a typical user. Search results were collected to approximate the content most likely encountered by a typical user. An initial set of 254 videos was retrieved through the TikTok search function, which represents a platform-generated preselection of the most relevant content related to spondylolisthesis. According to previous studies, TikTok typically restricts the number of displayed search results to approximately 200-300 videos, as users generally browse within this range (Zhang et al., 2025; Yeung et al., 2022; Chambers et al., 2024). After applying the predefined exclusion criteria (duplicate entries, non-English language, and lack of relevance to the topic), a total of 82 videos were included in the final analysis. The selection and exclusion process is illustrated in Fig. 1.

### 2.2 Data collection and content categorization

Videos meeting the inclusion criteria were analyzed for key metrics, including duration (seconds), total views, number of likes, and number of shares at the time of data collection. The uploader was classified into one of four categories: private user, physiotherapist, surgeon, or researcher. Private users were defined as accounts without verifiable professional credentials in the profile (username or biography) or disseminated content, and without any documented affiliation to a healthcare or academic institution. Identification of physiotherapists relied on explicit professional designations (e.g., “PT,” “Physiotherapist”) or a demonstrable connection to physiotherapy services. Surgeons were classified according to medical degrees (e.g., “MD,” “DO”), declaration of a surgical specialty, or institutional affiliation with a healthcare provider. Researchers encompassed individuals with institutional affiliations in academia or research laboratories, membership in research groups, or engagement in research-focused dissemination in the absence of clinical qualifications. Video content was categorized according to its primary focus into one of the following groups: patient experience, anatomy, injury mechanism, physical therapy, surgical technique, or clinical tests. Although each video was assigned a primary content category, thematic overlap (e.g., anatomy combined with physical therapy) was frequent. This was noted descriptively, and multivariate models accounted for dominant topic without excluding overlapping content.

### 2.3 Assessment of video reliability and educational quality

All included videos were independently evaluated by two orthopedic surgeons to determine their reliability and educational value. The interrater agreement was evaluated using the intraclass correlation coefficient (ICC), which demonstrated excellent reliability (>0.8) across all assessment tools. Three established and validated assessment tools were employed: the DISCERN questionnaire, the Journal of the American Medical Association (JAMA) benchmark criteria, and the Global Quality Score (GQS). These instruments have been widely applied in previous studies assessing the quality of online health information (Subramanian et al., 2023; Glover et al., 2025; Zhang et al., 2025).

The DISCERN instrument (https://www.discern.org.uk) is a validated 16-item questionnaire designed to assess the reliability and quality of written or multimedia health information. It is organized into three domains: items 1-8 evaluate reliability, items 9-15 assess the quality of information regarding treatment options, and item 16 provides an overall quality rating. Items 1-15 are scored on a 5-point scale (1 = inadequate, 5 = fully adequate), yielding a total score between 15 and 75. Item 16 serves as a global rating, with 1 indicating very poor and 5 indicating excellent quality. Total DISCERN scores are interpreted as follows: 16-26 = very poor, 27-38 = poor, 39-50 = fair, 51-62 = good, and 63-75 = excellent.

The JAMA benchmark criteria evaluate four key domains relevant to the transparency and credibility of online medical content: authorship, attribution, disclosure, and currency. One point is awarded for each domain that is satisfied, resulting in a total score ranging from 0 to 4. Scores of 0-1 reflect insufficient information, scores of 2-3 indicate partially sufficient information, and a score of 4 represents fully sufficient information.

The GQS provides an overall assessment of the educational value and usefulness of a video. It employs a 5-point scale, where a score of 1 denotes poor quality and limited utility, and a score of 5 reflects excellent quality with high educational relevance.

### 2.4 Statistics

Prior to further analysis, continuous variables were tested for normality using the Shapiro–Wilk test, and homogeneity of variances was assessed with Levene's test. As variables were normally distributed, descriptive data are reported as mean ± standard deviation (SD) and range. A multiple linear regression model was performed to identify independent predictors of DISCERN, JAMA, and GQS scores, including video length, engagement metrics (views, likes, shares), video source, and content category as covariates. Multicollinearity was assessed using the variance inflation factor (VIF), with values < 5 considered acceptable. Differences in scores according to video source and content type were analyzed using the Mann–Whitney *U* test. Due to small subgroup sizes, physiotherapists, physicians, and researchers were combined into a single “health care professional” category, and anatomy, physical therapy, and surgical technique videos were grouped as “educational content.” Statistical significance was defined as a p-value <0.05. All statistical analyses were performed using R (version 4.4.2; R Foundation for Statistical Computing, Vienna, Austria).

## 3 Results

In total, 82 TikTok videos related to spondylolisthesis were analyzed. Together, they accumulated 4,151,631 views, received 55,967 likes, and were shared 7919 times, with a total video duration of 5635 s. Detailed engagement metrics and video characteristics are summarized in Table 1.

**Table 1:** Video characteristics and engagement metrics.

Variable	Mean ± SD (Range)	Total (n = 82)
Views	49,291.85 ± 110,467.88 (130–591,765)	4,151,631
Likes	656.53 ± 1165.58 (15-6223)	55,967
Shares	86.73 ± 186.14 (0-1068)	7919
Video length [s]	69.22 ± 88.61 (6-541)	5635

**Fig. 2:**
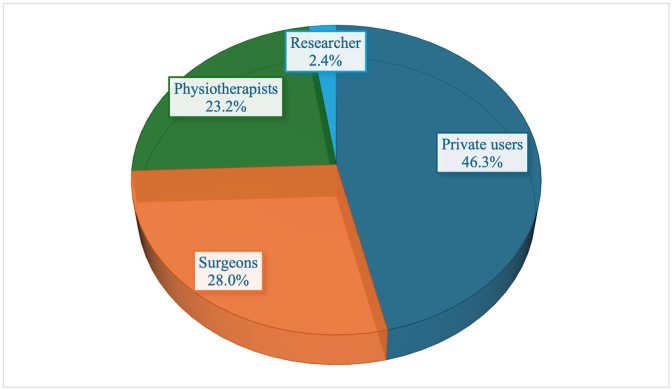
Uploader categories of spondylolisthesis-related TikTok videos.

Analysis of uploader revealed that private users were responsible for 46.3% of the content, followed by surgeons (28.0%), physiotherapists (23.2%), and researchers (2.4%; Fig. 2). Regarding content, videos frequently addressed multiple topics. Overall, 43 videos (53.1%) included anatomy-related information, 34 (42.0%) presented patient experiences, 26 (32.1%) focused on physical therapy, 22 (27.2%) discussed injury mechanisms, 14 (17.3%) demonstrated surgical techniques, and 7 (8.6%) covered clinical tests.

The mean DISCERN score across all videos was 34.14 ± 17.64, ranging from 10 to 78. The JAMA score averaged 1.76 ± 1.12 (range, 0–4), while the GQS reached 2.58 ± 1.10 (range, 1–5). Videos uploaded by health care professionals achieved significantly higher scores compared with those posted by private users for DISCERN (p < 0.001), GQS (p < 0.001), and JAMA (p < 0.001). Similarly, educational content scored significantly higher than patient experience videos for DISCERN (p < 0.001), GQS (p < 0.001), and JAMA (p < 0.001; Table 2).

**Table 2:** Comparison of DISCERN, global quality score (GQS), and JAMA benchmark scores by uploader type and content category.

Category	DISCERN (mean ± SD)	GQS (mean ± SD)	JAMA (mean ± SD)	p-value
Overall (n = 82)	34.14 ± 17.64	2.58 ± 1.10	1.76 ± 1.12	-
*Uploader*				
Private users	20.97 ± 9.08	1.76 ± 0.72	0.86 ± 0.67	<0.001
Health care professionals	45.74 ± 15.01	3.31 ± 0.84	2.55 ± 0.80	
*Content*				
Patient experience	19.68 ± 6.74	1.68 ± 0.59	0.91 ± 0.79	<0.001
Educational content	41.75 ± 15.39	3.05 ± 0.92	2.25 ± 0.92	

**Fig. 3:**
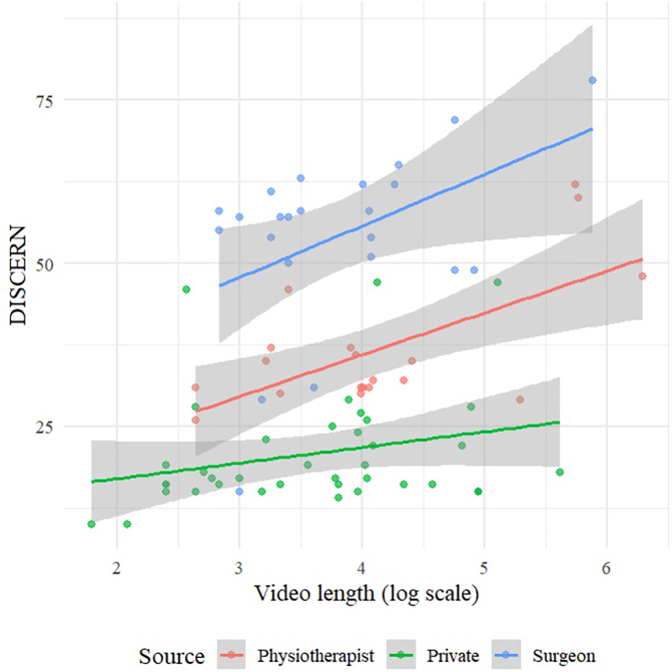
Association between video length (log scale) and DISCERN scores stratified by uploader type.

In the multivariate analysis longer video length was consistently associated with higher DISCERN (p < 0.001), GQS (p = 0.001), and JAMA scores (p = 0.006). Across all three measures, videos uploaded by surgeons (p < 0.001 for all scores) and physiotherapists (DISCERN: p = 0.003; GQS: p = 0.011; JAMA: p < 0.001) achieved significantly higher quality ratings compared with those from private users (Fig. 3). Regarding content type, anatomy- (DISCERN: p < 0.001; GQS: p = 0.003) and injury mechanism–related videos (DISCERN: p < 0.001; GQS: p < 0.001; JAMA: p = 0.016) were significantly associated with higher quality scores. Full multivariate results for DISCERN are shown in Table 3, with corresponding models for GQS and JAMA provided in Supplements 1 and 2.

**Table 3:** Independent predictors of TikTok DISCERN scores.

Variable	β (95%-CI)	p-Value	VIF
Video length [s]	0.051 (0.031; 0.070)	<0.001	1.06
*Engagement Metrics*			
Views	0.937 (−0.430; 2.303)	0.176	1.75
Likes	−1.328 (−3.498; 0.842)	0.226	2.22
Shares	1.733 (−0.253; 3.719)	0.086	2.24
*Uploader*			
Private users	Reference	Reference	1.39
Surgeons	19.904 (14.206; 25.602)	<0.001	1.39
Physiotherapists	10.011 (3.625; 16.397)	0.003	1.39
Researchers	0.668 (−15.325; 16.662)	0.934	1.39
*Type of Content*			
Patient experiences	Reference	Reference	1.40
Anatomy	15.728 (8.647; 22.809)	<0.001	1.40
Physical therapy	5.184 (−1.706; 12.074)	0.138	1.40
Injury mechanisms	15.072 (9.378; 20.767)	<0.001	1.40
Clinical tests	-	-	1.40

## 4 Discussion

This is the first study to systematically assess TikTok videos related to spondylolisthesis, demonstrating that the majority of videos offer low-quality information with limited reliability and educational benefit. The mean DISCERN score indicated “poor” quality, the mean JAMA score was rated as “insufficient,” and the mean GQS corresponded to “low educational value”. Almost half of the videos (46.3%) were posted by private users, while the rest originated from surgeons (28.0%), physiotherapists (23.2%), and researchers (2.4%). Videos created by healthcare professionals achieved significantly higher DISCERN, GQS, and JAMA scores than those produced by private users. Likewise, educational content scored significantly higher than patient experience videos across all three scoring systems.

Although no prior TikTok-based analysis focusing specifically on spondylolisthesis exists, a YouTube-based evaluation by Yaradılmış et al. demonstrated similarly poor reliability and educational value of videos related to this condition, with the majority of content failing to meet established quality benchmarks (Yaradılmış et al., 2020). This aligns closely with our findings, suggesting that the challenge of delivering accurate, evidence-based information about spondylolisthesis extends across multiple social media platforms.

Comparable observations have been reported in prior TikTok-based investigations of spine-related surgical content. In their assessment of low back pain videos, Hasan et al. identified a mean DISCERN score of 34, indicating poor overall quality and closely reflecting the findings of the present study (Hasan et al., 2024). Subramanian et al. evaluated the quality of spine surgery information on TikTok using the DISCERN tool and reported a mean score of 24.4, which is classified as “very poor quality” - even lower than the mean DISCERN score of 34.1 (“poor”) observed in the present study (Subramanian et al., 2023). Similarly, an evaluation of TikTok videos related to scoliosis by Chambers et al. revealed that the majority of the content was of very poor quality, with two of 220 videos being rated as fair and no videos receiving a good or very good rating (Chambers et al., 2024). In line with these findings, Glover et al. conducted a separate investigation of scoliosis-related TikTok content using both the DISCERN and GQS tools, likewise demonstrating generally poor quality and highlighting the widespread presence of misinformation on the platform (Glover et al., 2025). As outlined above, prior studies consistently demonstrated that the quality of spine-related TikTok content is poor. In contrast, analyses of diabetes- and Chronic Obstructive Pulmonary Disease (COPD)-related videos have reported markedly higher DISCERN ratings, indicating a substantially higher quality of information compared with spine surgery (Subramanian et al., 2023; Kong et al., 2021; Song et al., 2021). These findings suggest that, while content related to the spine appears to be particularly insufficient, the quality of medical information on TikTok varies depending on the specialty and topic.

Our analysis further demonstrated that nearly half of the TikTok videos addressing spondylolisthesis were uploaded by private users, whereas contributions from healthcare professionals, including surgeons and physiotherapists, accounted for only a minority. A similar distribution has been reported in prior TikTok-based analyses of spine-related topics, where non-professional creators were likewise identified as the dominant source of content (Subramanian et al., 2023; Glover et al., 2025; McBriar et al., 2023). This finding is consistent with the cross-sectional survey by Samtani et al. of 325 spine surgeons from 76 U.S. institutions, which revealed that despite nearly two-thirds having a professional presence on social media, inactivity was common, reflecting apprehensions about information quality, data security, and legal accountability. These barriers may account for the continued underrepresentation of healthcare professionals in the digital dissemination of spine-related knowledge (Samtani et al., 2023). In addition to this imbalance in content sources, our analysis showed that videos produced by healthcare professionals achieved significantly higher DISCERN, GQS, and JAMA scores than those uploaded by private users, highlighting the critical role of professional expertise in ensuring reliable and high-quality information. Comparable results have been reported in prior TikTok-based studies on musculoskeletal and spine-related topics, where videos created by healthcare professionals consistently outperformed those of laypersons in accuracy and educational value (Subramanian et al., 2023; Zhang et al., 2025; Hasan et al., 2024). In line with this finding, analysis of the present study's content type revealed that videos with an educational focus achieved higher scores than patient experience-based content across all scoring systems, emphasizing the added value of evidence-informed material over subjective narratives.

In addition to uploader and content type, previous investigations have highlighted video characteristics as relevant factors influencing quality. In their assessment of spine surgery content on TikTok, Subramanian et al. reported that video length was significantly linked to higher quality ratings (Subramanian et al., 2023). Zhang et al. further support this association, as their evaluation of osteoporosis-related TikTok videos demonstrated that longer video duration was linked to higher reliability and overall quality ratings (Zhang et al., 2025). Consistent with these observations, the present study likewise demonstrated that longer videos achieved higher DISCERN, GQS, and JAMA scores, indicating that adequate time may be necessary to convey more comprehensive educational content. Furthermore, our analysis demonstrated that engagement metrics such as views, likes, and shares had no measurable impact on content quality, indicating that popularity does not necessarily equate to educational value. Interestingly, Subramanian et al. even reported a reciprocal pattern, whereby videos with greater view counts were significantly more likely to exhibit lower quality scores (Subramanian et al., 2023).

Collectively, the findings of our evaluation align with the broader literature demonstrating that spine surgery-related content on social media is typically of low quality, with restricted reliability and limited educational value. Given TikTok's expanding influence as a platform for health-related information, the predominance of low-quality content represents a critical concern for patient education and highlights the urgent need to improve overall quality standards. Addressing this challenge will require greater engagement from healthcare professionals, with surgeons and physiotherapists in particular playing a central role in delivering accurate, evidence-based educational content. Importantly, our findings demonstrate that popularity metrics such as likes, shares, and views do not reflect informational accuracy, and patients should therefore not equate popularity with quality. Instead, more reliable information can be expected from longer, educationally focused videos created by healthcare professionals, rather than patient experience-based content from lay users. To translate these insights into practice, continuing medical education (CME) and professional development (CPD) programs, in collaboration with professional societies and healthcare institutions, could include dedicated modules that train healthcare professionals to produce evidence-based, high-quality social media content, communicate complex medical information effectively, and participate in digital education responsibly. Furthermore, verified healthcare channels, established through collaboration between professional societies and social media platforms, could serve as a trusted source of accurate medical information and enhance public confidence in online health education. These approaches may serve to normalize and professionalize clinicians' engagement on social media, reframing it as a meaningful extension of modern medical education and patient communication.

This study has several limitations. First, it focused exclusively on TikTok, whereas other social media platforms differ in video formats, user demographics, and content moderation policies, which limits the generalizability of our findings. Second, video inclusion may have been influenced by search and selection bias, as TikTok's proprietary algorithm is shaped by factors such as location, personalization, and trending content. Although a new account with no prior search history was used to minimize this effect, potential bias cannot be fully excluded. Additionally, unlike some other platforms, TikTok does not provide a validated method to verify content creator credentials, making it difficult to assess credibility with certainty. To increase reliability, categorical data such as uploader type were independently classified by two observers, but some degree of uncertainty remains. Moreover, the temporal nature of social media means that our findings represent only a snapshot, as content availability and trends evolve rapidly. Only English-language videos were analyzed, which may not reflect the quality of information available in other languages. Finally, although standardized and widely accepted scoring instruments were employed, video assessment inherently involves an element of subjectivity.

## 5 Conclusion

TikTok videos related to spondylolisthesis demonstrated low quality, limited reliability, and poor educational value, with nearly half of the content produced by private users rather than healthcare professionals. Although videos created by surgeons and physiotherapists, as well as longer, educationally focused contributions, achieved significantly higher DISCERN, JAMA, and GQS scores, overall standards remain insufficient for evidence-based patient education. Given the expanding role of social media in influencing patient knowledge and expectations, spine specialists and professional societies share a responsibility to provide accurate, accessible, and engaging content to improve digital health literacy and reduce the impact of low-quality information.

## Ethical approval

As this study exclusively analyzed publicly available educational videos on spondylolisthesis and did not involve human participants, patient data, or any intervention, institutional review board approval was not required.

## Authors’ contribution

All listed authors made substantial contribution to the research design, data collection or analysis, and the preparation of this manuscript.

CA: Measurement, Data curation, Formal Analysis, Supervision, review and editing.

ME: Conceptualization, Methodology, Data curation, Formal Analysis, Writing-original draft, review and editing.

LS: Data curation, Formal Analysis, Review and editing, Supervision.

LB: Review and editing, Supervision.

MM: Review and editing, Supervision.

FS: Review and editing, Supervision.

MP: Review and editing, Supervision.

TF: Conceptualization, Methodology, Measurement, Data curation, Formal Analysis, Supervision, Visualization, Writing-original draft, review and editing.

## Data availability statement

The datasets generated and analyzed during the current study are available from the corresponding author upon reasonable request.

## Funding

The authors report no financial support or research grants were received in relation to the planning, execution, or writing of this study.

## Declaration of competing interest

The authors declare that they have no known competing financial interests or personal relationships that could have appeared to influence the work reported in this paper.
