# Supplementary material for: Swipe, watch, learn? An analysis of TikTok as a source of patient education on spondylolisthesis
Source: Brain Spine. 2026 Feb 3;6:105960. doi: 10.1016/j.bas.2026.105960 (PMC12892042; doi:10.1016/j.bas.2026.105960)
Supplement: Multimedia component 1 [file mmc1.docx]

**SUPPLEMENT DATA:**

**Supplement Table 1.** Independent predictors of TikTok GQS scores

| **Variable** | **β (95%-CI)** | **p-Value** | **VIF** |
| --- | --- | --- | --- |
| Video length [s] | 0.003 (0.001; 0.004) | 0.001 | 1.06 |
| *Engagement Metrics* | | | |
| Views | 0.048 (-0.060; 0.155) | 0.380 | 1.75 |
| Likes | 0.060 (-0.111; 0.231) | 0.488 | 2.22 |
| Shares | 0.025 (-0.132; 0.181) | 0.752 | 2.24 |
| *Uploader* | | | |
| Private users | Reference | Reference | 1.39 |
| Surgeons | 1.077 (0.628; 1.526) | < 0.001 | 1.39 |
| Physiotherapists | 0.660 (0.157; 1.164) | 0.011 | 1.39 |
| Researchers | 0.900 (-0.360; 2.161) | 0.158 | 1.39 |
| *Type of Content* | | | |
| Patient experiences | Reference | Reference | 1.40 |
| Anatomy | 0.851 (0.293; 1.409) | 0.003 | 1.40 |
| Physical therapy | 5.184 (-1.706;12.074) | 0.138 | 1.40 |
| Injury mechanisms | 0.992 (0.544; 1.441) | < 0.001 | 1.40 |
| Clinical tests | - | - | 1.40 |

**Supplement Table 2.** Independent predictors of TikTok JAMA scores

| **Variable** | **β (95%-CI)** | **p-Value** | **VIF** |
| --- | --- | --- | --- |
| Video length [s] | 0.002 (0.001; 0.004) | 0.006 | 1.06 |
| *Engagement Metrics* | | | |
| Views | -0.001 (-0.112; 0.110) | 0.984 | 1.75 |
| Likes | -0.039 (-0.216; 0.137) | 0.659 | 2.22 |
| Shares | 0.111 (-0.050; 0.273) | 0.174 | 2.24 |
| *Uploader* | | | |
| Private users | Reference | Reference | 1.39 |
| Surgeons | 1.640 (1.177; 2.104) | < 0.001 | 1.39 |
| Physiotherapists | 1.084 (0.564; 1.603) | < 0.001 | 1.39 |
| Researchers | 0.870 (-0.431; 2.170) | 0.187 | 1.39 |
| *Type of Content* | | | |
| Patient experiences | Reference | Reference | 1.40 |
| Anatomy | 0.477 (-0.099; 1.053) | 0.103 | 1.40 |
| Physical therapy | -0.041 (-0.601; 0.519) | 0.885 | 1.40 |
| Injury mechanisms | 0.574 (0.111; 1.038) | 0.016 | 1.40 |
| Clinical tests | - | - | 1.40 |
